# Quercetin Is a Flavonoid Breast Cancer Resistance Protein Inhibitor with an Impact on the Oral Pharmacokinetics of Sulfasalazine in Rats

**DOI:** 10.3390/pharmaceutics12050397

**Published:** 2020-04-26

**Authors:** Yoo-Kyung Song, Jin-Ha Yoon, Jong Kyu Woo, Ju-Hee Kang, Kyeong-Ryoon Lee, Seung Hyun Oh, Suk-Jae Chung, Han-Joo Maeng

**Affiliations:** 1 College of Pharmacy, Seoul National University, Seoul 08826, Korea; yksong777@gmail.com; 2 Laboratory Animal Resource Center, Korea Research Institute of Bioscience and Biotechnology, Ochang 28116, Korea; Kyeongrlee@kribb.re.kr; 3 College of Pharmacy, Gachon University, Incheon 21936, Korea; jinha89@daum.net (J.-H.Y.); apoptosis@snu.ac.kr (J.K.W.); applekjh0503@hanmail.net (J.-H.K.); eyeball@gachon.ac.kr (S.H.O.)

**Keywords:** quercetin, breast cancer resistance protein, inhibitor, prazosin, sulfasalazine, kinetic analysis, pharmacokinetics, food–drug interactions

## Abstract

The potential inhibitory effect of quercetin, a major plant flavonol, on breast cancer resistance protein (BCRP) activity was investigated in this study. The presence of quercetin significantly increased the cellular accumulation and associated cytotoxicity of the BCRP substrate mitoxantrone in human cervical cancer cells (HeLa cells) in a concentration-dependent manner. The transcellular efflux of prazosin, a stereotypical BCRP substrate, was also significantly reduced in the presence of quercetin in a bidirectional transport assay using human BCRP-overexpressing cells; further kinetic analysis revealed IC_50_ and Ki values of 4.22 and 3.91 μM, respectively. Moreover, pretreatment with 10 mg/kg quercetin in rats led to a 1.8-fold and 1.5-fold increase in the AUC_8h_ (i.e., 44.5 ± 11.8 min∙μg/mL vs. 25.7 ± 9.98 min∙μg/mL, *p* < 0.05) and C_max_ (i.e., 179 ± 23.0 ng/mL vs. 122 ± 23.2 ng/mL, *p* < 0.05) of orally administered sulfasalazine, respectively. Collectively, these results provide evidence that quercetin acts as an in vivo as well as in vitro inhibitor of BCRP. Considering the high dietary intake of quercetin as well as its consumption as a dietary supplement, issuing a caution regarding its food–drug interactions should be considered.

## 1. Introduction

Flavonoids are a large group of polyphenolic antioxidants present in various human foods, such as vegetables, fruits, and tea. Quercetin is a major plant flavonol, a subclass of flavonoids with a 3-hydroxyflavone structure; it is present in high levels in onions, kale, broccoli, and tea [1,2]. Quercetin is mostly present in foods in the form of glycosides, which are efficiently hydrolyzed in the small intestine to release quercetin aglycone when ingested [3]. Dietary consumption of quercetin is estimated to be between 25 and 50 mg per day, accounting for approximately 70% of the total dietary flavonol and flavonone intake [4,5,6]. Moreover, it is well recognized that quercetin has diverse biological effects, including antioxidative, antiviral, antiulcer, and anticancer activities [7,8,9,10]. These activities have led to its consumption in various dosages and forms (e.g., 200–1000 mg aglycone per capsule/tablet [11]) as dietary supplements.

However, a recent analysis reported that the increased demand and consumption of dietary supplements is likely associated with a risk of adverse events. Indeed, a high number of adverse events (i.e., 23,000 emergency department visits per year in the United States) are attributed to dietary supplements [12]. In particular, flavonoids can modulate the activity of major ATP-binding cassette (ABC) efflux transporters [13]. For example, several studies have consistently shown that quercetin interacts with both P-glycoprotein (P-gp) [14,15,16] and multidrug resistance-associated protein 1 (MRP1) [17], inhibiting the efflux of substrates in the specific transporter-overexpressing cells in vitro or increasing the bioavailability/brain accumulation of substrate drugs in vivo by affecting the transporters’ activity. Moreover, our research group recently reported that repeated pretreatment with quercetin could upregulate the human multidrug resistance protein 1 (MDR1) gene via a vitamin D receptor-dependent pathway in Caco-2 cells [18]. Therefore, the increasing use of dietary supplements containing quercetin emphasizes the need to investigate the potential clinical interactions that can be induced by the flavonoid.

Among ABC transporters, breast cancer resistance protein (BCRP; encoded by the ABCG2 gene) is a major efflux transporter abundantly expressed at the apical membrane of intestinal/kidney epithelial cells and hepatocytes. The transporter functions as a physiological barrier against oral absorption as well as a determinant of the disposition of substrate drugs [19]. Recently, several studies have attempted to determine whether quercetin interacts with BCRP. Sesink et al. reported that the flavonoid can be transported by the mouse Bcrp1 transporter in MDCKII/mBcrp1 cells [20]; moreover, its presence was shown to inhibit the cellular accumulation of the BCRP substrates Hoechst 33342 and mitoxantrone in BCRP-overexpressing MCF-7 cells [21,22]. However, such observations in cell systems cannot be directly translated to substantial effects on efflux transporter activity in vivo. For example, a study reported that coadministration of topotecan (a BCRP substrate) with the flavonoids chrysin or 7,8-benzoflavone (potent inhibitors of the transporter in BCRP-overexpressing MCF-7 cells) resulted in no significant effects on the pharmacokinetics of the substrate in rats or P-gp-knockout mice [23]. Therefore, considering that no apparent in vitro to in vivo association regarding BCRP inhibition by flavonoids was found [23], a more pharmacokinetic-based understanding of the interaction of quercetin with BCRP is needed. To our knowledge, the in vivo pharmacokinetic inhibition of BCRP by quercetin has not been previously reported.

Therefore, the objective of this study was to conduct an integrated study including in vitro and in vivo pharmacokinetic assessments on the inhibition of BCRP by quercetin. Here, we showed that quercetin can increase the cellular accumulation and associated cytotoxicity of the BCRP substrate mitoxantrone in human cervical cancer HeLa cells. Importantly, the high inhibitory potency of quercetin in limiting transporter-mediated efflux was demonstrated using the kinetic parameters (e.g., IC_50_ and Ki) associated with the efflux. Finally, the in vivo pharmacokinetics of the possible inhibition were studied in rats using sulfasalazine, a selective BCRP probe that was previously proven to show increased absorption by impaired BCRP function [24,25,26].

## 2. Materials and Methods

### 2.1. Materials

**Figure 1:**
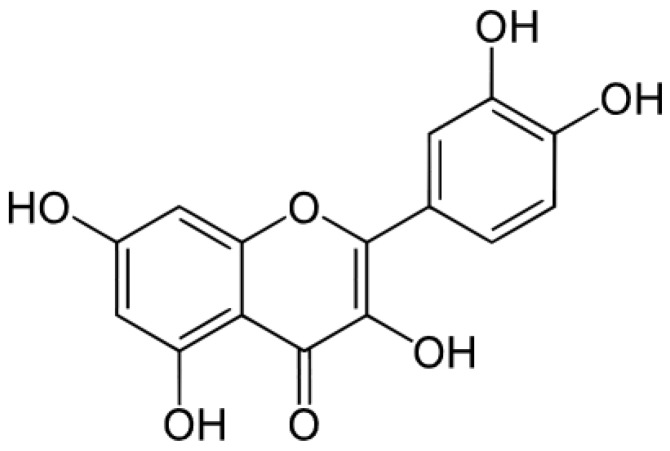
Chemical structure of quercetin.

Quercetin (Figure 1), mitoxantrone (MX), Ko143, and sulfasalazine were purchased from Sigma-Aldrich (St Louis, MO, USA). Prazosin was purchased from Tokyo Chemical Industry (Tokyo, Japan). High-performance liquid chromatography-grade methanol and formic acid were purchased from Fisher Scientific (Pittsburgh, PA, USA) and Fluka (Cambridge, MA, USA), respectively.

### 2.2. Cell Culture

For the cellular accumulation and cytotoxicity studies, HeLa (human cervical cancer) cells were cultured in Dulbecco’s modified Eagle’s medium (DMEM; Welgene Inc., Daegu, Korea) supplemented with 10% fetal bovine serum (FBS; Welgene Inc., Daegu, Korea) and 100 U/mL penicillin–100 μg/mL streptomycin at 37 °C in a humidified incubator with 5% CO_2_. For the bi-directional transport study, previously established human BCRP-overexpressing MDCKII cells [27] were used. Briefly, a plasmid construct containing cDNA for human BCRP was transfected into wildtype MDCKII cells to functionally express the transporter. MDCKII cells were grown in DMEM containing 10% FBS, 1% nonessential amino acid solution, 100 units/mL penicillin, and 0.1 mg/mL streptomycin under a humidified atmosphere containing 5% CO_2_ at 37 °C.

### 2.3. RT-PCR Analysis

To measure the gene expression levels at the RNA level of BCRP, reverse-transcription polymerase chain reaction (RT-PCR) was performed. Total RNA was isolated from Hela, Caco-2, MCF-7, and SW620 cells using TRIzol reagent (Invitrogen, Carlsbad, CA, USA); complementary DNA (cDNA) was synthesized from 2 μg of the RNA extracted from cells, using the PrimeScript RT reagent Kit (TaKaRa, Shiga, Japan). cDNA was then amplified by PCR using human-specific primers: BCRP, 5′-TTC TCC ATT CAT CAG CCT CG-3′ (forward) and 5′-TGGTTGGTCGTCAGGAAGA-3′ (reverse); GAPDH 5′-GAA GGT GAA GGT CGG AGT C-3′ and 5′-GAAGATGGTGATGGGATTTC-3′ (reverse). Reverse transcription PCR (RT-PCR) was performed in a T-100TM thermal cycler (Bio-Rad, Hercules, CA, USA) using AccuPower PCR Premix (Bioneer, Daejeon, Korea), according to the manufacturer’s protocol. The thermocycler conditions used for amplification were 95 °C for 5 min (hot start), 94 °C for 45 s, 55 °C for 30 s, and 72 °C for 30 s in 30 (BCRP) or 26 (GAPDH) cycles. Subsequently, the resultant products were analyzed by separation on a 1.5% agarose gel in tris-acetate/ethylenediaminetetraacetic acid (EDTA) buffer.

### 2.4. FACS-Cellular Accumulation Study

The cellular accumulation of quercetin was measured by FACSCalibur flow cytometry (Becton Dickinson, San Jose, CA, USA). For FACScan analysis, 2  ×  10^5^ HeLa cells/well were seeded into 6-well cell culture plates on the day before the experiment. On the following day, cells were treated with vehicle or quercetin and 1 μM MX. A time course experiment was conducted on HeLa cells following treatment with quercetin (1 and 100 μM) for 2, 4, and 6 h. After treatment, the cells were harvested by trypsinization and transferred to a fluorescence-activated cell sorting (FACS) tube, pelleted by centrifugation (1500 rpm, 5 min), and then resuspended in 200 μL of PBS. Flow cytometry analysis was performed using red fluorescence. A minimum of 10,000 cells were acquired per sample.

### 2.5. Cytotoxicity Assay

To determine the cytotoxic efficacy (i.e., the anticancer activity) of mitoxantrone associated with its intracellular accumulation, we performed the Cell Counting Kit-8 assay (CCK-8 assay kit; Dojindo Molecular Technologies, Kumamoto, Japan) following the manufacturer’s instructions. HeLa cells (at a density of 1 × 10^4^ cells per well) were seeded and cultured overnight in 96-well plates. Then, the medium was replaced with fresh medium containing the test drugs (mitoxantrone alone, mitoxantrone with 1 μM or 100 μM quercetin); the antiproliferation potential was examined at different drug concentrations after 24 h of incubation [28]. Additionally, 1 μM Ko143 was used as a positive control for BCRP inhibition. The absorbance was measured at a wavelength of 450 nm using a microplate reader (BioTeK, Highland Park, WI, USA).

### 2.6. Bi-Directional Transport Study

For the evaluation of the in vitro inhibitory potential of human BCRP by quercetin, the basolateral-to-apical (B-to-A) and apical-to-basolateral (A-to-B) permeability coefficients (P_app_) of prazosin (the stereotypical substrate of BCRP) were determined in BCRP-overexpressing MDCKII cells in the presence of various concentrations of quercetin. Briefly, MDCKII cells were seeded on Transwell^®^ filters (12 mm diameter, 0.4 μm pore size; Corning, NY, USA) at a density of 0.5 × 10^6^ cells∙mL^−^^1^ and then cultured for 5 days before being used in the transport assays. The confluence and integrity of the tight junctions were confirmed via microscopic observations as well as the measurement of transepithelial resistance [29]. The cells were washed twice and pre-incubated with transport buffer (9.7 g/L Hanks’ balanced salt solution, 2.38 g/L HEPES, and 0.35 g/L sodium bicarbonate, pH adjusted to 7.4) for 30 min at 37 °C. Transport was initiated by adding transport buffer containing 10 μM prazosin in the presence or absence of quercetin (in a final concentration range of 0.1–300 μM) to the donor compartment (500 μL for the apical chamber or 1.5 mL for the basolateral chamber), followed by incubation at 37 °C for 120 min. At the end of the incubation, aliquots (300 μL for the apical chamber and 500 μL for the basolateral chamber) of the incubation mixture were collected from the donor and receiver chambers and subjected to LC-MS/MS assays.

### 2.7. Experimental Animals

Eight male Sprague-Dawley rats weighing 230–270 g (Orient Bio Inc., Seongnam, Korea) were used in the in vivo studies. The experimental protocols involving animals were reviewed and approved by the Seoul National University Institutional Animal Care and Use Committee, according to the National Institutes of Health Principles of Laboratory Animal Care (publication number 85-23, revised in 1985). The animal protocol number was SNU-180521-4; this protocol was approved on 9 October 2018.

### 2.8. Oral Pharmacokinetic Study in Rats

To determine whether quercetin affects the intestinal efflux mediated by BCRP, we divided the male rats into two groups: A sulfasalazine (a substrate of BCRP) control group and a quercetin pretreatment plus sulfasalazine group (*n* = 4, each). Considering the similar expression levels of intestinal BCRP between male and female rats, male rats were used in this study [30,31]. Briefly, overnight fasted male SD rats were anesthetized by intramuscular administration of 50 mg/kg tiletamine HCl/zolazepam HCl (Zoletil^®^) (Vibrac, TX, USA) and 10 mg/kg xylazine HCl (Rompun^®^, Bayer, Puteaux, France). While the rats were anesthetized, the femoral artery (for blood sampling) and vein (for supplementing body fluids) were catheterized using polyethylene tubing (PE 50; Clay Adams, Parsippany, NJ, USA). Upon recovery from anesthesia (i.e., after 4 h), quercetin was administered by oral gavage at 10 mg/kg (or 0 mg/kg in the case of the sulfasalazine control group; DMSO/polyethylene glycol 400/saline [1:4:5 (v/v/v)]). The pretreatment dose of quercetin was determined based on the compound solubility in the dosing vehicle and the likely daily dose of human dietary supplement. Fifteen minutes after the pretreatment, a dosing solution containing sulfasalazine at 2 mg/kg was administered by oral gavage. Blood samples (150 μL) were collected at 5, 15, 30, 60, 120, 240, 360, and 480 min after the sulfasalazine administration. Immediately after each blood collection, an identical volume of saline was intravenously provided to the animal to compensate for fluid loss. To prevent blood clotting during blood collection, the cannula was filled with 25 IU/mL heparinized saline. The plasma fraction was separated from the blood samples by centrifugation (16,100 × g for 5 min at 4 °C) and stored at −80 °C until the LC-MS/MS assay.

### 2.9. Quantification Using LC-MS/MS

Chromatographic quantification of sulfasalazine and prazosin was carried out using an LC-tandem mass spectrometry (LC-MS/MS) system equipped with a Waters e2695 high-performance liquid chromatography system (Milford, MA, USA) and an API 3200 QTRAP mass spectrometer (Applied Biosystems, Foster City, CA, USA). Briefly, an aliquot (50 µL) of a sample was vortex-mixed with an acetonitrile solution containing glipizide (300 ng/mL, internal standard); this was followed by centrifugation (16,100 × g for 5 min at 4 °C). An aliquot (5 µL) of the supernatant was directly injected into the LC-MS/MS system. Separations were carried out using a gradient of 0.1% formic acid in acetonitrile and 0.1% formic acid in water at a flow rate of 0.7 mL/min using a reversed-phase high-performance LC column (Agilent Poroshell 120, EC-C18 2.7 µm, 4.6 × 50 mm). The following transitions were used for analyte detection: *m/z* 399.0 → *m/z* 380.8 for sulfasalazine and *m/z* 384.1 → *m/z* 95.0 for prazosin. For the internal standard glipizide, the transition *m/z* 445.8 → *m/z* 320.9 was used. The limits of quantification were 10 ng/mL for sulfasalazine and 50 nM for prazosin.

### 2.10. Data Analysis

#### 2.10.1. In Vitro Kinetic Analysis

The apparent permeability coefficient (P_app_) of prazosin was estimated using the following equation (Equation (1)):

(1) $$P_{app}=\frac{1}{A}\times \frac{1}{C_{0}}\times \frac{dQ}{dt}$$

where *dQ*/*dt*, A, and C_0_ represent the transport rate, the surface area of the insert, and the initial concentration of the compound in the donor compartment, respectively. The efflux ratio (ER) was calculated by dividing the B-to-A apparent permeability coefficient (P_app_, _B-to-A_) by the A-to-B apparent permeability coefficient (P_app_, _A-to-B_). In the inhibition studies, the percentage of the control efflux ratio (%ER) was also calculated by dividing the value for ER in the presence of the inhibitor by that in the absence of the inhibitor (i.e., in the control). When necessary, the half maximal inhibitory concentration (IC_50_) was determined by nonlinear regression analysis using WinNonlin Professional 5.0.1 software (Pharsight Corporation, Mountain View, CA, USA) and the following equation (Equation (2)):

(2) $$V=V_{max}-\left(V_{max}-V_{0}\right)\times \left[\frac{{\left[I\right]}^{n}}{{\left[I\right]}^{n}+{\left(IC_{50}\right)}^{n}}\right]$$

where V, V_max_, V_0_, [I], and n represent the rate of transport in the presence of the inhibitor, the maximal rate of transport, the basal rate of transport, the concentration of the inhibitor, and the Hill coefficient, respectively. When it was necessary to convert the IC_50_ to the inhibitory constant (Ki), the following equation (Equation (3)) [32] was used under the assumption that competitive inhibition existed between the substrate and the inhibitor:

(3) $$K_{i}=\frac{IC_{50}}{1+\frac{\left[S\right]}{K_{m}}}$$

where [S] is the concentration of the substrate and K_m_ represents the Michaelis–Menten constant.

#### 2.10.2. Non-Compartmental Pharmacokinetic Analysis

Standard non-compartmental pharmacokinetic analysis was carried out using WinNonlin Professional 5.0.1 software (Pharsight, Cary, NC, USA) to calculate the pharmacokinetic parameters, including the peak concentration (C_max_), time of the peak concentration (t_max_), elimination half-life (t_1/2_), area under the plasma concentration–time curve from time zero to the last sampling point, 8 h (AUC_8h_), and elimination clearance (CL/F).

### 2.11. Statistical Analysis

For the comparison of means among the groups, one-way ANOVA (analysis of variance; for cytotoxicity and bi-directional transport studies) followed by Tukey’s post hoc test were used. In these in vitro studies, a value of *p* < 0.05 was considered statistically significant. For the comparison of means between the groups for in vivo studies, the two-tailed/unpaired Student’s t-test was used and a value of *p* < 0.05 with a statistical power more than 0.8 (Minitab 19.2, Minitab Inc., State College, PA, USA) was considered statistically significant.

## 3. Results

### 3.1. FACS-Cellular Accumulation Study

**Figure 2:**
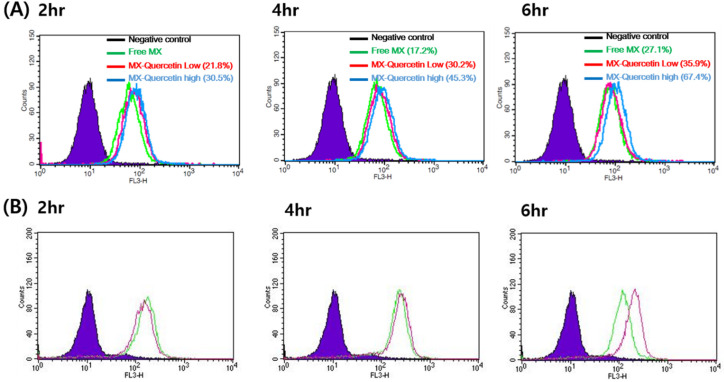
Representative histogram of mitoxantrone (MX) uptake in HeLa cells. (**A**) Flow cytometry measurement of MX fluorescence in HeLa cells incubated with MX alone (green line) or MX with 1 (red line) or 100 μM quercetin (blue line) for 2, 4, and 6 h. (**B**) Flow cytometry measurement of MX fluorescence in HeLa cells incubated with MX alone (green) or MX with 1 μM Ko143, a specific breast cancer resistance protein (BCRP) inhibitor (purple line), for 2, 4, and 6 h.

The expression of BCRP in Hela cells was confirmed by RT-PCR and compared with other cells, which were known to express high (Caco-2 and MCF-7) or low (SW620) levels of BCRP (Supplementary Figure S1) [33,34]. In the FACS-cellular accumulation study, the potential of quercetin to inhibit BCRP was first investigated by observing the cellular uptake of mitoxantrone (MX). The cellular uptake of MX with or without quercetin was analyzed by flow cytometry. The fluorescence intensity of a single cell measured by flow cytometry can be a good indication of the amount of MX internalized by each cell. As shown in Figure 2A, the peak fluorescence intensity of MX uptake was shifted to a higher level when MX was co-administered with quercetin, suggesting the promotion of MX internalization in HeLa cells. In the MX single treatment group, the percentage of cells with a significant uptake of MX was higher by 17.2% at 4 h of treatment and 27.1% at 6 h of treatment than at 2 h of treatment with MX alone as a control. In contrast, the cellular uptake of MX in the presence of quercetin was considerably higher by 30.2% at 4 h of treatment and 35.9% at 6 h of treatment (co-treatment with 1 μM quercetin) and by 45.3% at 4 h of treatment and 67.4% at 6 h of treatment (co-treatment with 100 μM quercetin) than at 2 h of treatment with MX alone. We also tested the internalization of MX when co-administered with 1 μM Ko143, a BCRP inhibitor. The results showed a considerably high number of cells that internalized MX when 1 μM Ko143 was co-administered with MX (Figure 2B). Thus, quercetin significantly promoted the cellular uptake of MX in HeLa cells likely via the inhibition of BCRP-mediated efflux.

### 3.2. Cytotoxicity of Mitoxantrone in the Presence of Quercetin

**Figure 3:**
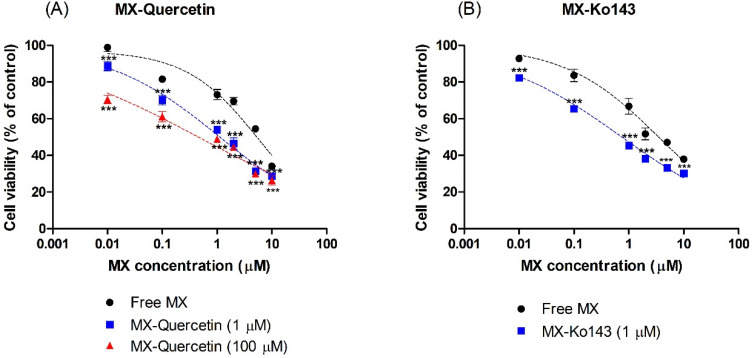
Effect of co-incubation of mitoxantrone (MX) with (**A**) quercetin (1 or 100 μM) and (**B**) Ko143 (1 μM) on the cell viability of HeLa cells. The Cell Counting Kit-8 (CCK-8) assay was used to determine the cytotoxicity associated with the cellular accumulation of MX after 24 h of incubation. Asterisks indicate statistical differences (* *p* < 0.05; ** *p* < 0.01; and *** *p* < 0.001) from the control group (i.e., without the quercetin or Ko143) according to one-way ANOVA, followed by Tukey’s post hoc test. Data are presented as the mean ± SD of quintuplicate runs.

To further confirm the effect of quercetin on the reversal of BCRP-mediated chemoresistance in HeLa cells, we examined the cytotoxicity (i.e., anticancer activity) of mitoxantrone in the absence and presence (1 or 100 μM) of quercetin. In this study, CCK-8 was used for the examination of mitoxantrone-associated cytotoxicity. As shown in Figure 3, mitoxantrone displayed concentration-dependent cytotoxicity in HeLa cells, which was further boosted in the presence of 1 μM Ko143, a stereotypical BCRP inhibitor. Likewise, the presence of 1 or 100 μM quercetin effectively enhanced the cytotoxicity associated with mitoxantrone as the IC_50_ decreased to 19.3% (1.13 μM) or 8.2% (0.478 μM), respectively, which differed from that observed with mitoxantrone alone (5.83 μM; Figure 3A). In addition, the cytotoxicity of quercetin alone without mitoxantrone was also examined. Treatment with 100 μM quercetin alone led to no significant changes in cell viability in comparison with the control (0.1% DMSO), demonstrating that the increased cytotoxicity observed in mitoxantrone-treated cells was not likely associated with the toxicity of quercetin (Supplementary Figure S2).

### 3.3. Bi-Directional Transport Study in MDCKII/BCRP Cells

**Figure 4:**
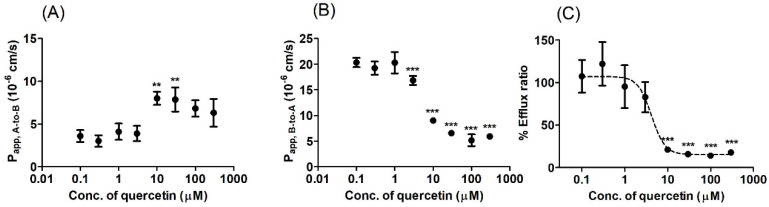
Bi-directional transport of prazosin in BCRP-overexpressing Madin-Darby Canine Kidney-II (MDCKII/BCRP) cells under various concentrations of quercetin (0.1–300 μM). (**A**) Apical-to-basolateral apparent permeability coefficient (P_app_, _A-to-B_) and (**B**) basolateral-to-apical apparent permeability coefficient (P_app_, _B-to-A_) of prazosin. (**C**) The percentage of the control efflux ratio (%ER, compared to the value without inhibitor) is shown together with the best-fit values generated from the nonlinear regression analysis based on Equation (2). Asterisks indicate statistical differences (* *p* < 0.05; ** *p* < 0.01; and *** *p* < 0.001) from the control (i.e., without quercetin) according to one-way ANOVA, followed by Tukey’s post hoc test. Data are presented as the mean ± SD of triplicate runs. Data are presented as the mean ± SD of triplicate runs.

**Figure 5:**
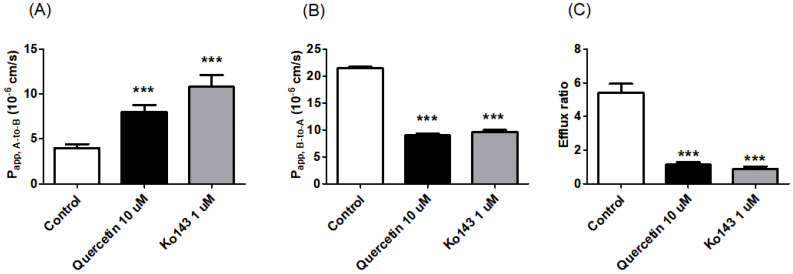
Effect of 10 μM quercetin or 1 μM Ko143 on the apparent permeability coefficient and efflux ratio of prazosin, a BCRP substrate, in MDCKII/BCRP cells. (**A**) Apical-to-basolateral apparent permeability coefficient (P_app_, _A-to-B_), (**B**) basolateral-to-apical apparent permeability coefficient (P_app_, _B-to-A_), and (**C**) efflux ratios of prazosin in the absence of inhibitor (i.e., the control) or in the presence of quercetin (10 μM) or Ko143 (the standard inhibitor of BCRP; 1 μM). Asterisks indicate statistical differences (* *p* < 0.05; ** *p* < 0.01; and *** *p* < 0.001) from the control group (i.e., without the inhibitor) according to one-way ANOVA, followed by Tukey’s post hoc test. Data are presented as the mean ± SD of triplicate runs.

We performed bi-directional transport studies in MDCKII cells expressing human BCRP (MDCKII/BCRP) to investigate the in vitro inhibitory potency of quercetin against BCRP in a concentration-dependent manner. Co-incubation with quercetin increased the P_app_, _A-to-B_ of prazosin (Figure 4A) while simultaneously decreasing the P_app_, _B-to-A_ (Figure 4B) with an increasing concentration of quercetin, leading to a concentration-dependent decrease in the overall ER (Figure 4C). Additionally, the functional expression of the efflux transporter in MDCKII/BCRP cells was also confirmed in this study, with an ER of 5.4 for prazosin (the stereotypical substrate of BCRP [27,35,36]), which decreased to 0.9 in the presence of the known inhibitor Ko143 (Figure 5C). Notably, the inhibitory effect of 10 μM quercetin on the B-to-A transport and efflux ratio was comparable to 1 μM Ko143 (Figure 5; *p* > 0.05). At quercetin concentrations higher than 10 μM, the ERs were less than 1.2, indicating the nearly complete inhibition of prazosin efflux (the complete inhibition of efflux would theoretically result in an ER of ~1, Figure 5). Kinetic analysis of the transport process yielded an estimated IC_50_ value of 4.22 μM for quercetin. Assuming the mechanism of inhibition to be competitive, the inhibitory constant (Ki) value was then estimated to be 3.91 μM using the K_m_ value of 128 μM [27] for prazosin.

### 3.4. Oral Pharmacokinetic Study in Rats with or without Quercetin

**Figure 6:**
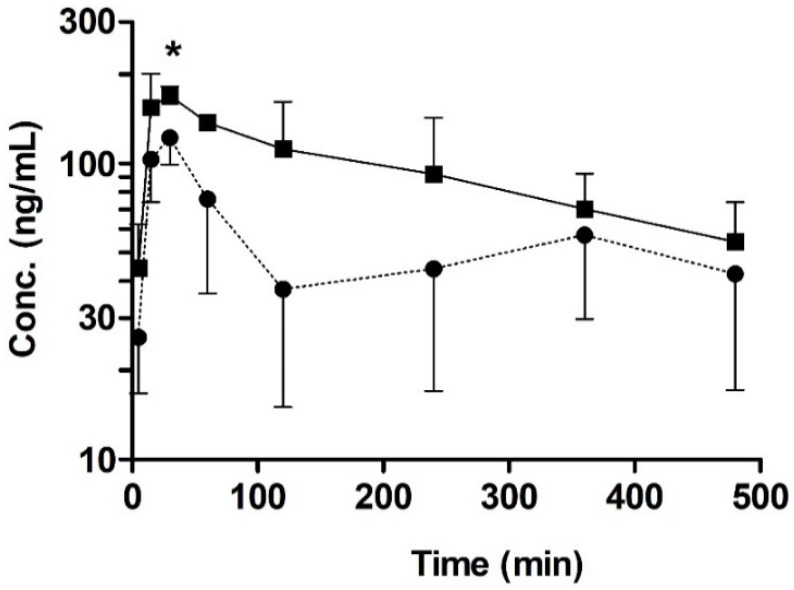
Temporal profiles of orally administered sulfasalazine (2 mg/kg) with or without the pre-administration of quercetin (10 mg/kg). Key: Control (●; without quercetin), quercetin pre-administration (■). Asterisks indicate statistical differences from the control (i.e., without quercetin) according to a two-tailed/unpaired Student’s t-test (* *p* < 0.05, power > 0.8). Data are expressed as the mean ± SD of quadruplicate runs.

To investigate the possible pharmacokinetic impact of quercetin as a BCRP inhibitor, we performed an oral pharmacokinetic study with sulfasalazine, a BCRP substrate, in rats. In this study, the change in the plasma concentration of sulfasalazine was used as an indicator of the in vivo interaction of BCRP with quercetin. To our knowledge, sulfasalazine has only limited interactions with other efflux transporters, including P-gp and MRP2 [34], whereas prazosin (the substrate used in the bi-directional transport study) is a dual substrate of P-gp and BCRP in vivo [37]. Thus, sulfasalazine is considered a relatively selective in vivo probe substrate of BCRP [25,26]. The mean plasma concentration–time profiles following the oral administration of 2 mg/kg sulfasalazine with or without pretreatment with 10 mg/kg quercetin in rats are shown in Figure 6. The pharmacokinetic parameters, as estimated using non-compartmental analysis, are summarized in Table 1. The plasma AUC_8h_ of sulfasalazine with or without quercetin pretreatment was 44.5 ± 11.8 min∙μg/mL and 25.7 ± 9.98 min∙μg/mL, respectively; this value was higher by 1.8-fold in the quercetin pretreatment group than in the control group, but it was not significantly different (*p* < 0.05, power < 0.8). More importantly, the C_max_ was significantly higher by 1.5-fold (*p* < 0.05, power > 0.8) in the quercetin pretreatment group (179 ± 23.0 ng/mL) than in the control group (i.e., 122 ± 23.2 ng/mL), whereas there was no significant change in the elimination half-life (t_1/2_) of sulfasalazine. Collectively, these results suggest that pretreatment with quercetin led to the increased oral absorption of sulfasalazine in vivo.

## 4. Discussion

Increasing lines of evidence from animal and human studies regarding food–drug interactions have indicated that a wide range of flavonoids can interact with ABC transporters, thereby leading to overexposure or underexposure of clinically important substrate drugs [13]. However, the accurate prediction of such interactions has been found to be difficult owing to limited in vitro data. The objective of this study was to investigate the inhibitory potential of quercetin against BCRP in vitro and in vivo. This study, which integrated the in vitro and in vivo effects of quercetin, was indeed necessary because a thorough understanding of the pharmacokinetic influence of this flavonoid is needed because of its high dietary intake as well as the lack of clear corresponding pharmacokinetic data.

Here, we demonstrated that the presence of quercetin can effectively enhance the cellular accumulation and associated cytotoxicity of mitoxantrone in HeLa cells (Figure 2 and Figure 3), consistent with previous reports [38]. In the current study, the efficacy of quercetin as a BCRP inhibitor was quantitively demonstrated via a significant reduction in the IC_50_ of mitoxantrone even in the presence of quercetin at a concentration as low as 1 μM (i.e., it decreased to 19.3% of the control value; from 5.83 to 1.13 μM). When the concentration of quercetin increased to 100 μM, the IC_50_ of mitoxantrone was further decreased (i.e., to 8.23% of the control value; 0.48 μM), similar to that in the presence of Ko143 (i.e., 0.62 μM), a stereotypical BCRP inhibitor. In addition, pharmacokinetically relevant parameters were obtained in a bi-directional transport study using MDCKII/BCRP cells, where the IC_50_ values of quercetin for the inhibition of BCRP-mediated efflux were estimated to be 4.22 μM. Assuming the mechanism of inhibition to be competitive, the IC_50_ value was further transformed to a Ki value of 3.91 μM, using the K_m_ value of 128 μM [27] for prazosin. The values obtained in the bi-directional transport studies were comparable to those previously observed in MCF-7/MX and MDCKII/BCRP cells using Hoechst 33342 accumulation (IC_50_ values of 7.6 and 6.9 μM, respectively) [21]. In both assays, it was shown that while quercetin is a less potent inhibitor compared to Ko143, it can show a similar inhibitory effect compared to 1 μM Ko143 in higher concentrations (Figure 3 and Figure 5).

The US Food and Drug Administration recommends that orally administered compounds with an [I_gut_] value (the maximal gastrointestinal concentration; defined as the dose divided by 250 mL) divided by the Ki value greater than 10 be evaluated for potential in vivo interactions [36]. For quercetin, the estimated [I_gut_] value (662 μM, assuming a dietary quercetin intake of 50 mg/day) or even the estimated intestinal concentration (86.2 μM, when the intestinal fluid volume is assumed to be 1.92 L [39]) divided by the Ki (3.91 μM) value is far greater than 10. Thus, although the bioavailability of quercetin is somewhat low [40] and the daily dietary intake reportedly results in sub-micromolar concentrations in circulation [3], the substantially higher concentration in the gut is likely to result in the inhibition of intestinal BCRP and thereby an increase in the intestinal absorption of BCRP transporter substrates.

Consequently, the in vivo inhibitory potency of quercetin was further assessed to clarify its interaction with intestinal BCRP. In this study, the pharmacokinetic profile of orally administered sulfasalazine was used as an indicator of any alterations in intestinal BCRP activity. While sulfasalazine has been reported to be effluxed by P-gp and MRP2 to a low extent, previous studies have consistently demonstrated that the intestinal absorption of the compound following its oral administration was essentially unaffected in P-gp- or MRP2-knockout rats in contrast to the significantly higher AUC_8h_ and C_max_ values observed in BCRP-knockout rats [24], strongly suggesting that sulfasalazine is a good probe for observing intestinal BCRP activity. In this study, higher AUC_8h_ and C_max_ values of sulfasalazine (1.8-fold (*p* < 0.05, power < 0.8) and 1.5-fold (*p* < 0.05, power > 0.8), respectively) were observed in the presence of 10 mg/kg quercetin than in its absence (Table 1). The increased absorption in the presence of quercetin is clearly significant, but the degree is somewhat lower than that expected considering the approximately 20-fold increase observed in knockout rats [24] and, especially, the low Ki value of the flavonoid obtained in the current study. One possible reason for this discrepancy might be the rapid conjugation of quercetin to quercetin-3-glucuronide that occurs in the small intestine [20]. Once quercetin enters the intestinal cells by passive diffusion or uptake by the uptake transporters, it is subjected to glucuronidation by a UDP-glucuronosyltransferase present in both rat and human intestines [41,42,43], which results in the rapid clearance of quercetin from sites adjacent to the efflux transporter. Indeed, the oral bioavailability of quercetin was only 5.3% and the C_max_ value was the sub-micromolar range (i.e., 0.21 µg/mL) following 10 mg/kg oral administration to rats [44]. Another possibility that might result in relatively limited alterations in sulfasalazine absorption is the involvement of OATP2B1 in the intestinal absorption of sulfasalazine [25]. Sulfasalazine is a high-affinity substrate of OATP2B1 [25,45], whereas quercetin has been reported to be an inhibitor of OATP2B1 [46]. Therefore, the relatively low increase in sulfasalazine exposure in the presence of quercetin may be attributed to complex interactions between the simultaneous inhibition of the efflux by BCRP and the uptake by OATP2B1. In addition, considering that we only observed a single dosing of quercetin on the sulfasalazine pharmacokinetics, further studies regarding multiple dosing of quercetin are likely needed.

In a previous study by Zhang et al., an apparent discrepancy between the in vitro and in vivo inhibition of BCRP by the flavonoids chrysin and 7,8-benzoflavone was reported. In their investigation, the flavonoids were demonstrated to be potent inhibitors of human BCRP but weak inhibitors of mouse BCRP [23]; one possible explanation for this discrepancy may be species differences between the human and rodent transporters. Although a further study regarding food–drug interaction is required in humans, this may also be true for quercetin, in which case the clinical impact of the modulation of BCRP activity in humans may be much greater than that estimated from pharmacokinetic studies performed in rats.

## 5. Conclusions

The in vitro and in vivo inhibitory potencies of quercetin against BCRP were examined focusing on functional and/or kinetic aspects. Quercetin significantly increased the cellular accumulation and associated cytotoxicity of mitoxantrone in HeLa cells in a concentration-dependent manner. The transcellular efflux of prazosin was significantly reduced in the presence of quercetin as observed in a bi-directional transport assay using MDCKII/BCRP cells. These modulations in BCRP activity were consistent with the in vivo results, where pretreatment with quercetin led to not very dramatically different but still significantly higher intestinal absorption of sulfasalazine compared to that in the control group. Collectively, these results provide evidence that quercetin acts as a potent inhibitor of BCRP both in vitro and in vivo. Considering the high dietary intake of quercetin as well as its consumption as a dietary supplement, careful attention should be paid to potential flavonoid–drug interactions.

## Figures and Tables

**Table 1:** Pharmacokinetic parameters of sulfasalazine after its oral administration (2 mg/kg dose) with and without pretreatment with quercetin (10 mg/kg) in rats. Data are expressed as the mean ± SD (*n* = 4 per group).

Parameter	Control	Pre-Administration Group (10 mg/kg Quercetin)
t_1/2_ (min)	383 ± 111	242 ± 80.7
t_max_ (min)	30 ± 0	22.5 ± 8.70
C_max_ (ng/mL)	122 ± 23.2	179 ± 23.0 *
AUC_8h_(min∙ng/mL)	25700 ± 9980	44500 ± 11800
CL/F (mL/min/kg)	52.5 ± 33.5	33.2 ± 10.2

* significantly different from the control (i.e., without the pre-administration of quercetin) (*p* < 0.05, power > 0.8).

## Supplementary Materials

The following are available online at https://www.mdpi.com/1999-4923/12/5/397/s1, Figure S1: mRNA expression levels of BCRP in HeLa, Caco-2, MCF-7 and SW620 cell lines using RT-PCR, Figure S2: Effect of 100 μM quercetin alone on the cell viability of HeLa cells.
