# Supplementary material for: Quercetin Is a Flavonoid Breast Cancer Resistance Protein Inhibitor with an Impact on the Oral Pharmacokinetics of Sulfasalazine in Rats
Source: Pharmaceutics. 2020 Apr 26;12(5):397. doi: 10.3390/pharmaceutics12050397 (PMC7285070; doi:10.3390/pharmaceutics12050397)
Supplement: Supplementary file 1 [file pharmaceutics-12-00397-s001.pdf]

pharmaceutics

Supplementary Materials: Quercetin is a Flavonoid Breast Cancer Resistance Protein Inhibitor with an Impact on the Oral Pharmacokinetics of Sulfasalazine in Rats

Yoo-Kyung Song, Jin-Ha Yoon, Jong Kyu Woo, Ju-Hee Kang, Kyeong-Ryoon Lee, Seung Hyun Oh, Suk-Jae Chung and Han-Joo Maeng

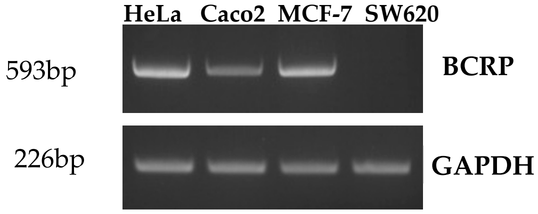

Figure S1. mRNA expression levels of BCRP in HeLa, Caco-2, MCF-7 and SW620 cell lines using RT-PCR. The RT-PCR was performed for 48 h-cultured HeLa, MCF-7 and SW620 cells and for 144 h-cultured Caco-2 cells after seeding the cells (2×10^5 cells/well) into 6-wells culture plates

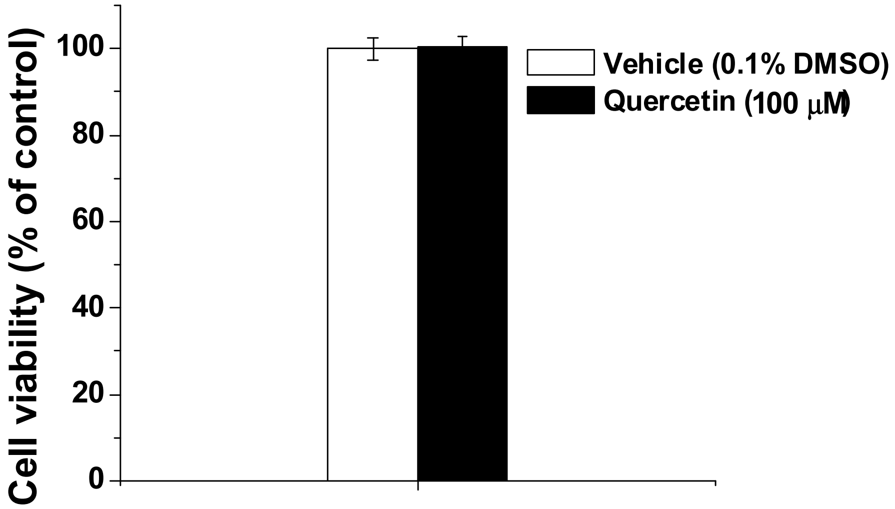

Figure S2. Effect of 100 μM quercetin alone on the cell viability of HeLa cells. CCK-8 toxicity test was used to determine the cytotoxicity associated with cellular accumulation of mitoxantrone. Data are presented as mean ± S.D. of triplicate runs.
